# Two‐Photon Polymerization of Nanocomposites for Additive Manufacturing of Transparent Magnesium Aluminate Spinel Ceramics

**DOI:** 10.1002/advs.202307175

**Published:** 2024-03-17

**Authors:** Richard Prediger, Nitipoom Sriyotha, Karl G. Schell, Sebastian Kluck, Leonhard Hambitzer, Frederik Kotz‐Helmer

**Affiliations:** ^1^ Laboratory of Process Engineering NeptunLab Department of Microsystems Engineering (IMTEK) University of Freiburg 79110 Freiburg Germany; ^2^ Institute for Applied Materials (IAM) Karlsruhe Institute of Technology (KIT) 76131 Karlsruhe Germany; ^3^ Freiburg Materials Research Center (FMF) University of Freiburg 79104 Freiburg Germany; ^4^ Glassomer GmbH In den Kirchenmatten 54 79110 Freiburg Germany

**Keywords:** additive manufacturing, hot isostatic pressing, sintering, transparent ceramics, transparent spinel, two‐photon lithography

## Abstract

Transparent polycrystalline magnesium aluminate (MAS) spinel ceramics are of great interest for industry and academia due to their excellent optical and mechanical properties. However, shaping of MAS is notoriously challenging especially on the microscale requiring hazardous etching methods. Therefore, a photochemically curable nanocomposite is demonstrated that can be structured using high‐resolution two‐photon lithography. The printed nanocomposites are converted intro transparent MAS by subsequent debinding, sintering, and hot isostatic pressing. The resulting transparent spinel ceramics exhibit a surface roughness *S_q_* of only 10 nm and can be shaped with minimum feature sizes of down to 13 µm. This technology will be important for the production of microstructured ceramics used for optics, photonics, or photocatalysis.

## Graphical abstract

Photochemically curable nanocomposites allow high‐resolution 3D printing of transparent magnesium aluminate ceramics using two‐photon lithography. After 3D printing, the nanocomposites are thermally debinded and fully densified by sintering and hot isostatic pressing. This allows the fabrication of complex structured transparent magnesium aluminate spinel  ceramics that are difficult to access by conventional methods.

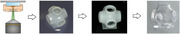

## 1 Introduction

Transparent magnesium aluminate (MgAl_2_O_3_) spinel (MAS) ceramics are of great interest due to their excellent mechanical and optical properties.^[^ ^1^ ^]^ Besides a hardness of up to 16 GPa and a melting point of 2135 °C, they demonstrate high chemical stability and a transmission of up to 87 % in the range of 250–5000 nm.^[^ ^2^, ^3^ ^]^ As a result of their cubic crystal structure, polycrystalline spinel ceramics are optically isotropic and therefore do not exhibit birefringence.^[^ ^4^ ^]^

Due to their superior material properties MAS ceramic are currently used for the production of simple geometries such as infrared domes or sensor windows.^[^ ^4^, ^5^ ^]^ Since they also combine a high refractive index *n_532 nm_* of 1.719 with a low dispersion (Abbe number ≈60), MAS ceramics would also be an interesting material for optical components such as thin lenses.^[^ ^1^, ^6^ ^]^ As a high refractive index is always associated with high dispersion in polymers and glasses, MAS ceramics thus have a significant advantage.^[^ ^7^ ^]^ The high transmittance combined with high thermal and chemical stability also proves useful as photocatalyst support.^[^ ^8^ ^]^ Since the efficiency depends on the surface area of the catalyst, microstructuring to achieve a high surface area is particularly required.^[^ ^9^ ^]^ Unfortunately, transparent MAS ceramics are notoriously difficult to shape especially on the microscale.

Transparent MAS ceramics are usually produced by methods such as hot pressing or slip casting followed by sintering and hot isostatic pressing (HIP).^[^ ^10^, ^11^ ^]^ However, these methods are mainly suitable for simple structures like disks or domes and post‐processing steps such as grinding or polishing are required to achieve optical surfaces.^[^ ^3^, ^11^, ^12^ ^]^ Recently, our group has shown that the production of transparent MAS is also possible via powder injection molding.^[^ ^13^ ^]^ Injection molding is well suited for the production of ceramics in high volumes. However, new tools are required for each new geometry, making low‐volume production uneconomical. Furthermore, the demand for complex 3D microstructured parts is increasing, which can't be shaped via molding.^[^ ^14^, ^15^, ^16^ ^]^ Recently, first 3D printing methods of transparent MAS have been demonstrated. For example, direct 3D printing via laser direct deposition has been shown, however the manufactured parts all showed prevalent cracking.^[^ ^17^ ^]^ Furthermore, stereolithography of transparent MAS starting from a nanocomposite was demonstrated, which for the first time was capable of fabricating truly 3D‐dimensional MAS parts with a resolution of a few 100 µm.^[^ ^8^ ^]^ However, due to the layer‐by‐layer based printing, the method is not capable of printing optical surfaces.

## 2 High‐Resolution 3D Printing of MAS Nanocomposites

In recent years, two‐photon lithography (TPL) has become a promising method for high‐resolution 3D microprinting. TPL uses a laser that is focused on a very confined region, leading to a multiphoton absorption at the very fine voxel, and allowing an extremely high printing accuracy.^[^ ^18^, ^19^ ^]^ It has already been shown that TPL can be used to print complex shapes from polymers with an extremely high precision and resolution.^[^ ^20^, ^21^, ^22^ ^]^ In addition, it has previously been shown that structuring of inorganic materials such as metals, non‐transparent ceramics as well as transparent glass‐ceramics, and glasses is feasible.^[^ ^23^, ^24^, ^25^, ^26^, ^27^, ^28^ ^]^ In 2006, Pham et. al. showed that SiCN ceramics can be structured using TPL with a resolution of up to 210 nm starting from a polymer photoresist, which acts as a precursor.^[^ ^29^ ^]^ The polymer photoresist was printed in high resolution and then converted into SiCN by thermal treatment. Based on this method, further processes have been developed that allow metals such as platinum or tungsten to be fabricated using TPL.^[^ ^30^, ^31^ ^]^ In addition, 3D printing of transparent silicate glass ceramics containing zirconium oxide was demonstrated.^[^ ^32^ ^]^ Fused silica, which could be fabricated starting from nanocomposites, has also already been presented as a transparent inorganic material for TPL.^[^ ^26^ ^]^ Transparent yttrium aluminum garnet is so far the only reported transparent non‐silicate ceramic that has been printed by TPL using a sol–gel approach.^[^ ^33^ ^]^ However transparency could only be shown for parts thinner than 1 µm due to a relatively high defect density in the parts. The materials further showed a high shrinkage of up to 68 % leading to deformations during the heat treatment process.^[^ ^33^ ^]^

Here, we describe for the very first time the fabrication of complex shaped microstructured MgAl_2_O_4_ nanocomposites using TPL, which can subsequently be converted into transparent MAS ceramics, with a feature size of down to 13 µm and a surface roughness *S_q_* of only 10 nm. The printed samples show a Vickers hardness of 1226 ± 70 HV and a transmission approaching the theoretical maximum.

**Figure 1:**
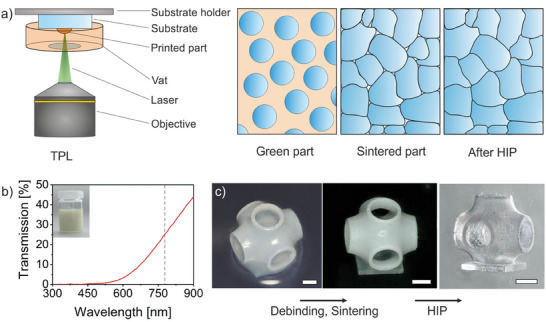
Fabrication of transparent MAS ceramics by TPL. a) Schematic illustration of the process for the production of transparent MAS ceramics via TPL. The part is printed on a substrate that is immersed in a vat containing the nanocomposite using TPL. The resulting green part consists of nanoparticles that are fixed in position by the organic binder matrix. After thermal removal of the binder matrix, a brown part is obtained, which then can be sintered to closed porosity and subsequently fully densified by HIP into a transparent MAS ceramic. b) Transmission spectrum of a 0.3 mm layer of the MAS ceramic nanocomposite in the range of 300–900 nm. A maximal transmission of 26.1 % was determined at the illumination wavelength of 780 nm. c) The process demonstrated on the example of a single Schwarz P cell. A green part, a debinded and sintered part as well as a transparent MAS ceramic after HIP are shown (scale bars, 100 µm).

The nanocomposites consist of MgAl_2_O_4_ nanoparticles with an average particle size in the range of 430 nm (Figure S1, Supporting Information) dispersed in a matrix of a monomeric binder mixture and the acidic dispersant 2‐[2‐(2‐methoxyethoxy)ethoxy]acetic acid (MEEAA). MEEAA was added to prevent agglomeration of the particles and reduce the viscosity of the material.^[^ ^8^ ^]^ Agglomerates moreover prevent homogeneous densification of the ceramics during subsequent sintering, which is mandatory for achieving full‐density and transparent parts.^[^ ^11^, ^34^ ^]^ Further, the binder contained a photoinitiator suitable for TPL. 3D printing was carried out on a commercial TPL printer type NanoOne (UpNano GmbH). Suitable printing parameters were determined as demonstrated in Figure S9 (Supporting Information). Excess non‐polymerized nanocomposite was removed by a development step in a solvent mixture. The organic binder matrix of the printed parts was removed by thermal debinding at 600 °C. Afterward, the so‐called brown parts were sintered at 1550 °C to polycrystalline ceramics with a density >97 % demonstrating a closed porosity. The correlation between densification and thickness is shown in Figure S5 (Supporting Information) for samples with a thickness of 250  and 500 µm (Supporting Information). The pre‐densified ceramics were further fully densified via HIP at 1700 °C and 1500 bar. The overall process is shown in **Figure** **1**a.

Figure 1b shows a transmission of 26.1 % at the processing wavelength of 780 nm of a 0.3 mm layer, which was sufficiently high to selectively cure the nanocomposite. Furthermore, the viscosity of the nanocomposite with a solid loading of 28 vol.% was analyzed (Figure S2, Supporting Information). Despite the relatively high viscosity of ≈38 Pa s (shear rate of 10 s^−1^), the nanocomposite could still be poured and processed. To investigate the stability of the nanocomposites, both the optical appearance and viscosity were studied over a period of 30 days. It was found that the nanocomposites do not show agglomeration after 30 days and the viscosity hardly changes. It can therefore be expected that the composites can be used over a period of several weeks. Figure 1c shows the process from a printed green part to a transparent MAS ceramic with an exemplary printed Schwarz P structure.

## 3 Sintering and HIP of 3D Printed MAS Nanocomposites

**Figure 2:**
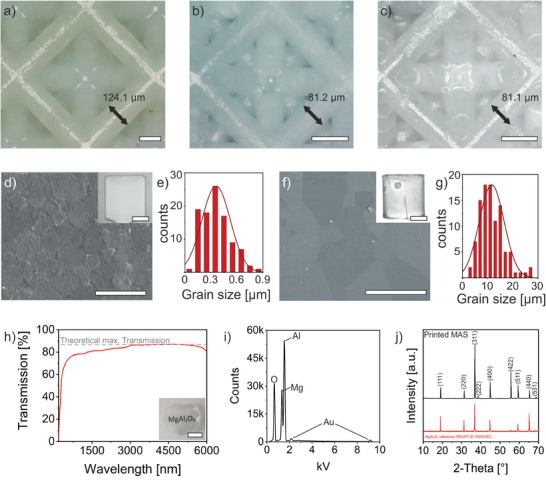
Characterization of MAS ceramics after sintering and HIP treatment. a) Green part having struts with a diameter of 124.1 µm. b) Sintered part having struts with a diameter of 81.2 µm. c) Part after HIP with a strut diameter of 81.1 µm (Scale bars a–c, 100 µm). An overall linear shrinkage of 34.65 % was determined. d) SEM image of the surface of a MAS ceramic (thickness: 300 µm) that was sintered for 2 h at 1550 °C (scale bar, 1 µm). The inset shows a sintered MAS ceramic (scale bar, 200 µm). e) Grain size distribution of a sintered MAS ceramic with a median grain size of 0.36  ± 0.16 µm. f) SEM image of a sample (thickness: 300 µm) after HIP for 2 h at 1700 °C and 1500 bar (scale bar, 1 µm). The inset shows a part after HIP (scale bar, 200 µm). g) Grain size distribution of MAS ceramics after HIP with median grain size of 11.58  ± 4.92 µm (scale bar, 20 µm). h) Transmission measurement of a MAS ceramic with a layer thickness of 250 µm after HIP. The dashed line indicates the maximum theoretical transmission of 87 % (scale bar inset, 1 cm). i) EDX‐measurement to analyze the elemental composition of MAS ceramics after HIP. Signals for gold are caused by the sputter layer. An elemental composition of ≈15 % Mg, 32 % Al, and 53 % O was determined. j) XRD measurement of a printed MAS ceramic after HIP. A reference diffractogram was added for comparison (RRUFF ID: R050392).

The printed green parts were thermally debinded and sintered. A detailed description of the debinding and sintering procedure can be found in Figure S3a (Supporting Information). During sintering to an almost complete density (>97 %) at 1550 °C the parts shrink isotropically by 34.57 ± 0.06 % (see **Figure** **2**a,b). This corresponds to a theoretical density of 99.9 % (see Equation S1, Supporting Information). A density of >97% after sintering is important to ensure closed porosity of the ceramics that is a requirement for HIP.^[^ ^13^ ^]^ It has been found that structures with a thickness of up to 500 µm can be debinded and sintered crack‐free (Figure S4, Supporting Information). After HIP, a total shrinkage of 34.65 ± 0.08 % was determined (see Figure 2c). This is in good agreement with the theoretical value of 34.58 % for a fully dense part.

Grain growth during different sintering stages was investigated by scanning electron microscopy (SEM) on polished and thermally etched samples (Figure 2d–g). A mean grain size of 0.36  ± 0.16 µm was determined after 2 h at 1550 °C. Furthermore, no abnormal grain growth was observed. Small grains favor complete densification of the ceramics, as remaining pores can be removed more successfully.^[^ ^35^ ^]^ During the HIP process, grains grew to a final mean grain size of 11.58  ± 4.92 µm. No abnormal grain size growth was observed during this step as well. After HIP the samples showed a dark discoloration, which has been described in literature as an oxygen vacancy happening during sintering.^[^ ^36^, ^37^ ^]^ This effect could be removed by thermally post‐treating the samples for 30 min at 1200 °C in atmospheric conditions (see Figure S3b, Supporting Information).

Additionally, the transmission of a sintered and HIPed MAS ceramic with a thickness of 250 µm was measured in the range from 190  to 6000 nm (Figure 2h). In the range from 190 to 1000 nm, the transmission approaches the theoretical maximum of 87%^[^ ^38^ ^]^ with an increasing wavelength. For wavelengths >1000 nm, a transmission >80% is achieved. At wavelengths from 3000  to 5000 nm the transmission reaches the theoretical maximum, while it decreases at higher wavelengths. Figure S9 (Supporting Information) shows that the transmission spectra of MAS samples with a thickness of 250 , 300  and 400 µm do not vary significantly from each other in the UV–vis and IR range. Energy dispersive X‐ray (EDX) measurements were conducted to investigate the elemental composition of the final MAS ceramics (see Figure 2i). The analyzed sample consisting of ≈15 % Mg, 32 % Al and 53 % O is in good agreement with the theoretical composition of MgAl_2_O_4_. Furthermore, a homogeneous distribution of the elements within the ceramic was observed (Figure S6, Supporting Information). X‐ray diffraction (XRD) analysis was performed and its results compared with a reference diffractogram (Figure 2j). It was confirmed that the crystal structure of the transparent ceramic consists of pure MAS. Additionally, the Vickers hardness of the transparent MAS ceramics was determined. The measured mean value of 1226 ± 70 HV or 12.02 ± 0.69 GPa (see Table S1, Supporting Information) is within the range of values from 12 to 16.8 GPa^[^ ^11^ ^]^ reported in the literature. Furthermore, the surface roughness of a printed planar structure was investigated. A surface roughness *S_q_* of only 10 nm (10 × 10 µm) was determined using WLI (see Figure S7a, Supporting Information). For a more extended area of 40 × 40 µm, an increased surface roughness *S_q_* of 26 nm was determined (see Figure S7b, Supporting Information).

**Figure 3:**
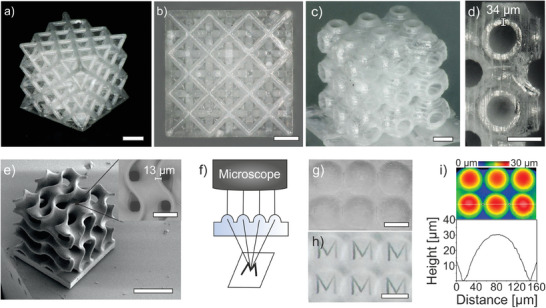
Printed transparent MAS ceramics after sintering and HIP. a) And b) octet lattice consisting of struts with a diameter of ≈80 µm (scale bars, 200 µm). c) And d) Schwarz P 3 × 3 × 3 array with a wall thickness of 34 µm (scale bars, 200 µm). e) SEM image of a gyroid structure after sintering and HIP (scale bar, 200 µm). The inset shows a feature size of only 13 µm. (scale bar, 50 µm). f) Scheme for the demonstration of the functionality of a microlens array by focusing through the lenses on an “M” below the printed part. g) SEM image of the micro lens array after HIP and h) the demonstrated functionality of the lenses according to the scheme in f) (scale bars g,h, 100 µm). i) WLI measurement of the printed microlens array in g).

**Figure** **3** shows various structures that were printed and converted into transparent ceramics using the method described. Figure 3a,b shows a microlattice with a strut diameter of ≈80 µm. A Schwarz P array with a wall‐thickness of 34 µm can be seen in Figure 3c,d. The gyroid structure in Figure 3e shows a single feature size of only 13 µm, being significantly smaller than previously shown stereolithography prints with 100 µm feature size.^[^ ^8^ ^]^ Furthermore, the functionality of printed transparent MAS ceramics was demonstrated in Figure 3f–h. The magnification effect could be shown through the lenses using an exemplary letter “M”. Using a white light interferometry (WLI), a surface roughness *R_q_* of 90.88 nm (100 µm profile) was shown on an exemplary printed lens (Figure 3i). The higher roughness can be explained by the vertically elongated voxel. This results in an inconsistent light exposure dose for curvatures and leads to an increased surface roughness.^[^ ^39^ ^]^

## 4 Conclusion

In summary, we demonstrate for the very first time high‐resolution 3D printing of transparent MAS ceramics using TPL of nanocomposites. Using this approach, structuring of MAS ceramics with a resolution of up to 13 µm and a surface roughness of only 10 nm is feasible, thus taking a major step toward optical quality. The printed components show a high optical transmission close to the theoretical maximum as well as high hardness of 12 GPa. The so far unprecedented resolution of TPL of MAS ceramics in combination with a high freedom of design leads to new opportunities for applications in optics, photonics, and functional surfaces.

## 5 Experimental Section

### Materials

All materials were used as supplied. MAS nanopowder S30CR was purchased from Baikowski, France. The acrylate‐based photochemical binder matrix including the photoinitiator bis(diethylamino)benzophenone was provided by Glassomer GmbH, Germany. 2‐[2‐(2‐Methoxyethoxy)ethoxy]acetic acid (MEEAA) was purchased from Sigma–Aldrich, Germany. Glassomer Developer was thankfully provided by Glassomer GmbH, Germany. The substrates used for printing were purchased from UpNano GmbH, Austria.

### Analysis of MAS Nanoparticles

The particle size distribution of the MAS nanoparticles was analyzed using a LS 13 320 laser diffraction particle size analyzer (Beckman Coulter, USA).

### Preparation of MAS Nanocomposite

For the preparation of the MAS nanocomposite, MEEAA was added to the binder and the mixture was stirred for 10 min at room temperature. Subsequently, the MAS nanopowder was added in small portions over a period of ca. 30 min, and the mixture was stirred using a laboratory stirrer type RZR 2102 (Heidolph Instruments GmbH & Co. KG, Germany) for additional 30 min at room temperature with a stirring speed of 1500 rpm. Before use, the nanocomposite was degassed in vacuum.

### Two‐Photon Lithography

3D printing using TPL was performed on a NanoOne printer (UpNano GmbH, Austria) with a 10x objective. The 3D structures were printed on substrates of fused silica (10 mm × 10 mm). Printing was performed using the following parameters: Profile: 10x Fine, print mode: Vat, slice mode: Simple, infil: Fine, Δxy = 1 µm, and Δz = 3 µm. The power of the laser ranged from 100 to 150 mW and a scanning speed between 400 and 600 mm s^−1^ was adjusted. This corresponds to an absorbed dose of 2–2.5 mJ cm^−1^. The fluence *F* was 516 J m^−^^2^. The laser worked with pulse lengths of 100 fs and a pulse frequency of 80 MHz. This results in 100 × 10^−13^ s pulses at a time interval of 1.25 × 10^−8^ s. It can therefore be assumed that no pulse overlap occurred. The printed parts were developed on the substrate in the Glassomer Developer, a mixture of solvents optimized for developing composite materials. For development, the printed parts were hung upside down in the developer at room temperature and developed for 15–30 min. Subsequently, the parts were dried for at least 1 h at room temperature. Afterward, the printed parts were detached from the substrate to allow isotropic shrinkage. As shown in Figure S10 (Supporting Information), the printing time increases significantly with the size of the parts. For this reason, only parts in the size range of up to a few millimeters were printed. 3D printing without photoinitiator was not possible with a dose of 2–7.5 mJ cm^−1^ using the same parameters as described before. At 6 mJ cm^−1^ and higher, bubbles started to form in the resin.

### Heat Treatment

The organic binder matrix of the green parts was removed in an ashing furnace of type AAF (Carbolite Gero, Germany). Afterward, the debinded parts were sintered to a density >97 % in a high‐temperature furnace type BLF 18/3 (Carbolite Gero, Germany) with a heating rate of 5 °C min^−1^ to a maximum temperature of 1550 °C and a dwell time between 1 and 3 h, depending on the thickness of the printed parts.

### Density Measurement

Density measurement was performed on samples with a diameter of 3 cm that have been produced by UV‐casting. The density (ρ) was measured using the Archimedes principle with a lab scale type Quintix 124‐1S and a density kit analytical balance YDK03 (Satorius AG, Germany). The pre‐sintered and HIP‐treated MAS ceramics were first measured in their dry state (*m*) and subsequently placed in DI water (T  =  22 °C). The density was then calculated using the following equation with *m_b_* being the buoyancy mass and $\rho_{H_{2}O}$ the density of water. The results can be seen in Figure S5 (Supporting Information).

(1) $$\rho =\rho_{H_{2}O}\times \frac{m}{m_{b}}$$

### Hot Isostatic Pressing

Complete densification of the pre‐sintered parts was performed in a hot isostatic press type QIH‐6 (ASEA, USA). The samples were treated in an argon atmosphere for 2 h at 1700 °C and 1500 bar. The heating and cooling rate was 10°C min^−1^. Several samples subsequently exhibited a slight discoloration, which was removed by an additional heat treatment of 30 min at 1200 °C in atmospheric conditions.

### Optical Characterization

The transmission of the nanocomposite as well as the fully densified MAS ceramics were measured using a UV–vis spectrometer type Evolution 201 (Thermo Scientific, Germany). Additionally, the transmission measurement in IR‐range was performed using a Fourier transform infrared (FTIR) spectrometer of type Frontier 100 MIR‐FTIR (Perkin Elmer, Germany). The samples were prepared by UV‐casting between two glass slides using a UV light source of type Superlite 400 (Lumatec, Germany) operating at a wavelength of 300–400 nm.

### Characterization of MAS Ceramic

XRD measurements were carried out in Bragg‐Brentano geometry with a D8 DISCOVER diffractometer (Bruker, Germany) equipped with Cu‐Kα radiation source and a LYNXEYE XE‐T detector. Measurements were performed with a step size of 0.05 ° in a 2θ span of 10–70 °. EDX measurements were performed with a spectrometer type Octane Elite EDS system (EDAX, Germany) with an acceleration speed of 20 kV. For the study of grain sizes, the samples were thermally etched by heating to a temperature of 1380 °C for 30 min after grinding and polishing. Subsequently, grain size analysis was performed on a SEM type Quanta FEG 250 (FEI Instruments, USA). The analysis of the roughness and the surface of the lens array of the MAS ceramics was performed using a White‐light interferometer type NewView 9000 (Zygo, USA). Vickers hardness was measured using a MHT‐10 microindentation tester (Anton Paar Germany) with a load of 100 kp and a load duration of 1 s. The mean value was determined from ten individual measurements.

## Conflict of Interest

The company Glassomer GmbH has patented the technology described within this paper (application no. PCT/EP2022/058116) and is in the process of commercializing it.

## Supporting information

Supporting Information

## Data Availability

The data that support the findings of this study are available in the supplementary material of this article.

## References

[1] A. Krell , T. Hutzler , J. Klimke , J. Eur. Ceram. Soc. 2009, 29, 207.

[2] G. J. Pereira , K. Bolis , D. N. F. Muche , D. Gouvêa , R. H. R. Castro , J. Eur. Ceram. Soc. 2017, 37, 4051.

[3] I. Ganesh , G. Jaganatha Reddy , G. Sundararajan , S. M. Olhero , P. M. C. Torres , J. M. F. Ferreira , Ceram. Int. 2010, 36, 473.

[4] S. F. Wang , J. Zhang , D. W. Luo , F. Gu , D. Y. Tang , Z. L. Dong , G. E. B. Tan , W. X. Que , T. S. Zhang , S. Li , L. B. Kong , Prog. Solid State Chem. 2013, 41, 20.

[5] Z. Xiao , S. Yu , Y. Li , S. Ruan , L. B. Kong , Q. Huang , Z. Huang , K. Zhou , H. Su , Z. Yao , W. Que , Y. Liu , T. Zhang , J. Wang , P. Liu , D. Shen , M. Allix , J. Zhang , D. Tang , Mater. Sci. Eng., R 2020, 139, 100518.

[6] S. S. Ramisetty , U. Kashalikar , M. Goldman , N. Nag , Am. Ceram. Soc. Bull 2013, 92, 20.

[7] P. Hartmann , R. Jedamzik , S. Reichel , B. Schreder , Appl. Opt. 2010, 49, D157.

[8] H. Wang , L. Y. Liu , P. Ye , Z. Huang , A. Y. R. Ng , Z. Du , Z. Dong , D. Tang , C. L. Gan , Adv. Mater. 2021, 33, 2007072. 10.1002/adma.202007072 33682251

[9] G. Tian , H. Fu , L. Jing , B. Xin , K. Pan , J. Phys. Chem. C 2008, 112, 3083.

[10] A. A. Kachaev , D. V. Grashchenkov , Y.. E. Lebedeva , S. St. Solntsev , O. L. Khasanov , Glass Ceram. 2016, 73, 117.

[11] M. Rubat du Merac , H.‐J. Kleebe , M. M. Müller , I. E. Reimanis , J. Am. Ceram. Soc. 2013, 96, 3341.

[12] A. Goldstein , A. Krell , J. Am. Ceram. Soc. 2016, 99, 3173.

[13] M. Mader , R. Prediger , K. G. Schell , G. Schmidt , A. Dorn , S. Jenne , S. Kluck , L. Hambitzer , M. Luitz , C. Schwarz , M. Milich , C. Greiner , B. E. Rapp , F. Kotz‐Helmer , Adv. Sci. 2022, 9, 2204385. 10.1002/advs.202204385 PMC9631057 36057994

[14] Z. Chen , Z. Li , J. Li , C. Liu , C. Lao , Y. Fu , C. Liu , Y. Li , P. Wang , Y. He , J. Eur. Ceram. Soc. 2019, 39, 661.

[15] Z. Chen , X. Sun , Y. Shang , K. Xiong , Z. Xu , R. Guo , S. Cai , C. Zheng , J. Adv. Ceram. 2021, 10, 195.

[16] E. Feilden , C. Ferraro , Q. Zhang , E. García‐Tuñón , E. D'Elia , F. Giuliani , L. Vandeperre , E. Saiz , Sci. Rep. 2017, 7, 13759. 29062036 10.1038/s41598-017-14236-9 PMC5653810

[17] J. M. Pappas , X. Dong , Materials 2020, 13, 4810. 33126542 10.3390/ma13214810 PMC7663333

[18] Q. Geng , D. Wang , P. Chen , S.‐C. Chen , Nat. Commun. 2019, 10, 2179. 31097713 10.1038/s41467-019-10249-2 PMC6522551

[19] M. Thiel , J. Fischer , G. von Freymann , M. Wegener , Appl. Phys. Lett. 2010, 97, 221102.

[20] T. Gissibl , S. Thiele , A. Herkommer , H. Giessen , Nat. Photonics 2016, 10, 554. 10.1038/ncomms11763 PMC4931017 27339700

[21] D. W. Yee , M. L. Lifson , B. W. Edwards , J. R. Greer , Adv. Mater. 2019, 31, 1901345. 10.1002/adma.201901345 PMC8063598 31231919

[22] M. Schumann , T. Bückmann , N. Gruhler , M. Wegener , W. Pernice , Light Sci. Appl. 2014, 3, e175.

[23] J. C. Sänger , B. R. Pauw , H. Sturm , J. Günster , Open Ceram. 2020, 4, 100040.

[24] A. Vyatskikh , S. Delalande , A. Kudo , X. Zhang , C. M. Portela , J. R. Greer , Nat. Commun. 2018, 9, 593. 29426947 10.1038/s41467-018-03071-9 PMC5807385

[25] A. Vyatskikh , R. C. Ng , B. Edwards , R. M. Briggs , J. R. Greer , Nano Lett. 2020, 20, 3513. 32338926 10.1021/acs.nanolett.0c00454

[26] F. Kotz , A. S. Quick , P. Risch , T. Martin , T. Hoose , M. Thiel , D. Helmer , B. E. Rapp , Adv. Mater. 2021, 33, 2006341. 10.1002/adma.202006341 PMC11469267 33448090

[27] F. Kotz , P. Risch , D. Helmer , B. E. Rapp , Adv. Mater. 2019, 31, 1805982. 10.1002/adma.201805982 30773705

[28] G. Balčas , M. Malinauskas , M. Farsari , S. Juodkazis , Adv. Funct. Mater. 2023, 33, 2215230.

[29] T. A. Pham , D.‐P. Kim , T.‐W. Lim , S.‐H. Park , D.‐Y. Yang , K.‐S. Lee , Adv. Funct. Mater. 2006, 16, 1235.

[30] M. Luitz , D. Pellegrini , M. von Holst , K. Seteiz , L. Gröner , M. Schleyer , M. Daub , A. Warmbold , Y. Thomann , R. Thomann , F. Kotz‐Helmer , B. E. Rapp , Adv. Eng. Mater. 2023, 25, 2201927.

[31] M. Luitz , M. Lunzer , A. Goralczyk , M. Mader , S. Bhagwat , A. Warmbold , D. Helmer , F. Kotz , B. E. Rapp , Adv. Mater. 2021, 33, 2101992. 10.1002/adma.202101992 PMC11469048 34337801

[32] K. Galvanauskas , D. Astrauskyte , G. Balcas , D. Gailevičius , L. Grineviciute , M. Malinauskas , 2023, 3, 20. 10.3390/nano13162281 PMC10458567 37630866

[33] I. Cooperstein , S. R. K. C. Indukuri , A. Bouketov , U. Levy , S. Magdassi , Adv. Mater. 2020, 32, 2001675. 10.1002/adma.202001675 32419262

[34] A. Krell , T. Hutzler , J. Klimke , A. Potthoff , J. Am. Ceram. Soc. 2010, 93, 2656.

[35] K. Tsukuma , J. Ceram. Soc. Jpn. 2006, 114, 802.

[36] A. Witek , Materiały Ceramiczne 2013, 65, 386.

[37] A. Goldstein , J. Eur. Ceram. Soc. 2012, 32, 2869.

[38] A. F. Dericioglu , Y. Kagawa , J. Eur. Ceram. Soc. 2003, 23, 951.

[39] X. Zhou , Y. Hou , J. Lin , AIP Adv. 2015, 5, 030701.

