# Supplementary material for: Two‐Photon Polymerization of Nanocomposites for Additive Manufacturing of Transparent Magnesium Aluminate Spinel Ceramics
Source: Adv Sci (Weinh). 2024 Mar 17;11(20):2307175. doi: 10.1002/advs.202307175 (PMC11132026; doi:10.1002/advs.202307175)
Supplement: Supplementary file 1 — Supporting Information [file ADVS-11-2307175-s001.pdf]

ADVANCED SCIENCE
Open Access

Supporting Information

for *Adv. Sci.*, DOI 10.1002/advs.202307175

Two-Photon Polymerization of Nanocomposites for Additive Manufacturing of Transparent Magnesium Aluminate Spinel Ceramics

*Richard Prediger, Nitipoom Sriyotha, Karl G. Schell, Sebastian Kluck, Leonhard Hambitzer and Frederik Kotz-Helmer**

Supporting Information

Two-Photon Polymerization of Nanocomposites for Additive Manufacturing of Transparent Magnesium Aluminate Spinel ceramics

*Richard Prediger, Nitipoom Sriyotha, Karl G. Schell, Sebastian Kluck, Leonhard Hambitzer, Frederik Kotz-Helmer**

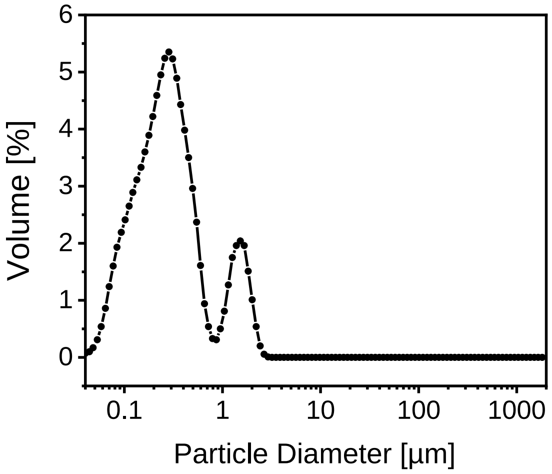

Figure S1. Analysis of the particle size distribution of the MAS nanoparticles used via static light scattering. The measurement shows that the main part of the particles has a diameter smaller than 1 µm. However, another fraction of the particles is in the range of 1 to 3 µm.

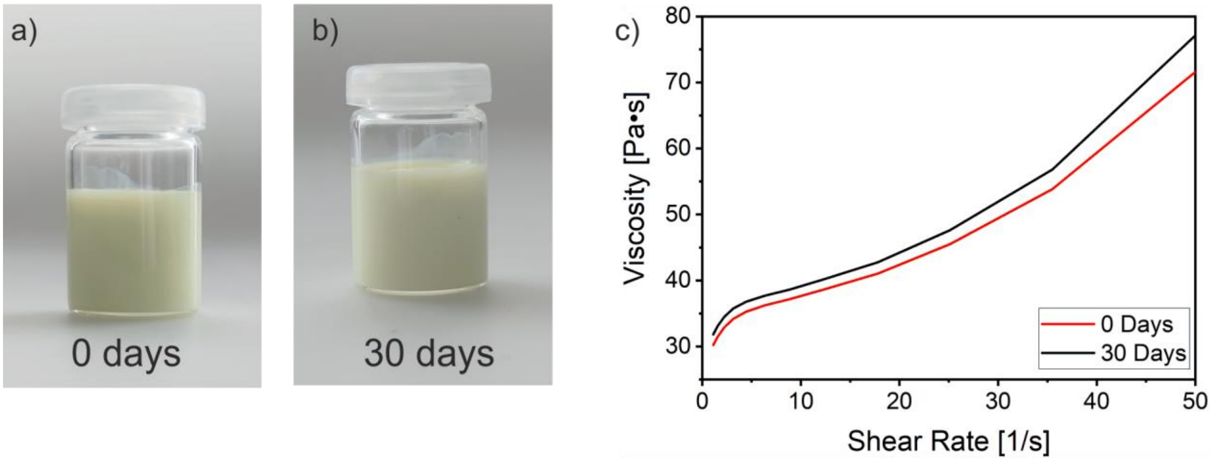

Figure S2. Analysis of the shelf life of the MAS nanocomposites. a) Image of a just produced nanocomposite. b) Image of the same nanocomposite after 30 days of storage at room temperature. Both images show an identical appearance. c) Viscosity measurements of the two nanocomposites after preparation and after storage for 30 days. The viscosity after 30 days is only slightly increased.

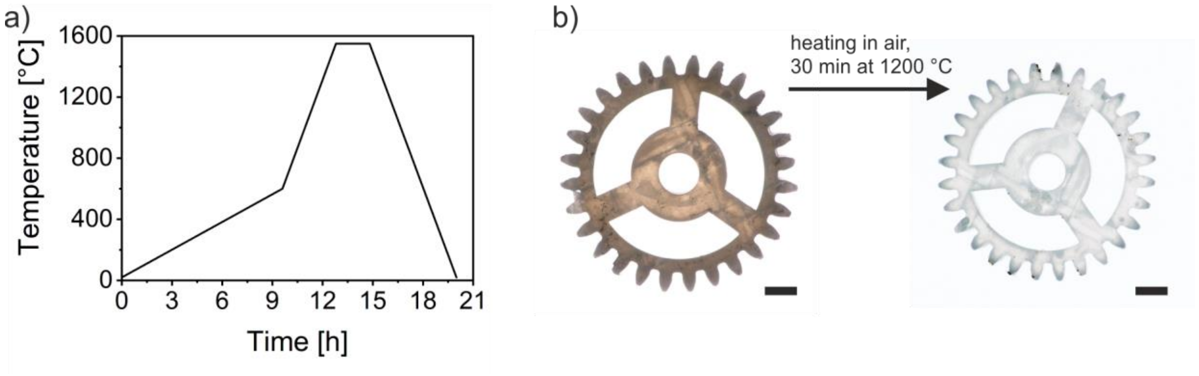

Figure S3. Thermal treatment of printed MAS nanocomposites and ceramics. a) Debinding and sintering protocol for the printed samples. First, debinding is performed with a heating rate of 1 °C/min up to a temperature of 600 °C. For sintering, the heating rate was then increased to 5 °C/min and the furnace heated to 1550 °C. After a dwell time of 2 h, the furnace cools to room temperature with a rate of 5 °C/min. b) Heat treatment of MAS samples after sintering and HIP to remove discoloration (scale bars, 100 µm). Heating in air to 1200 °C for 30 min removes the dark discoloration of the MAS ceramics.

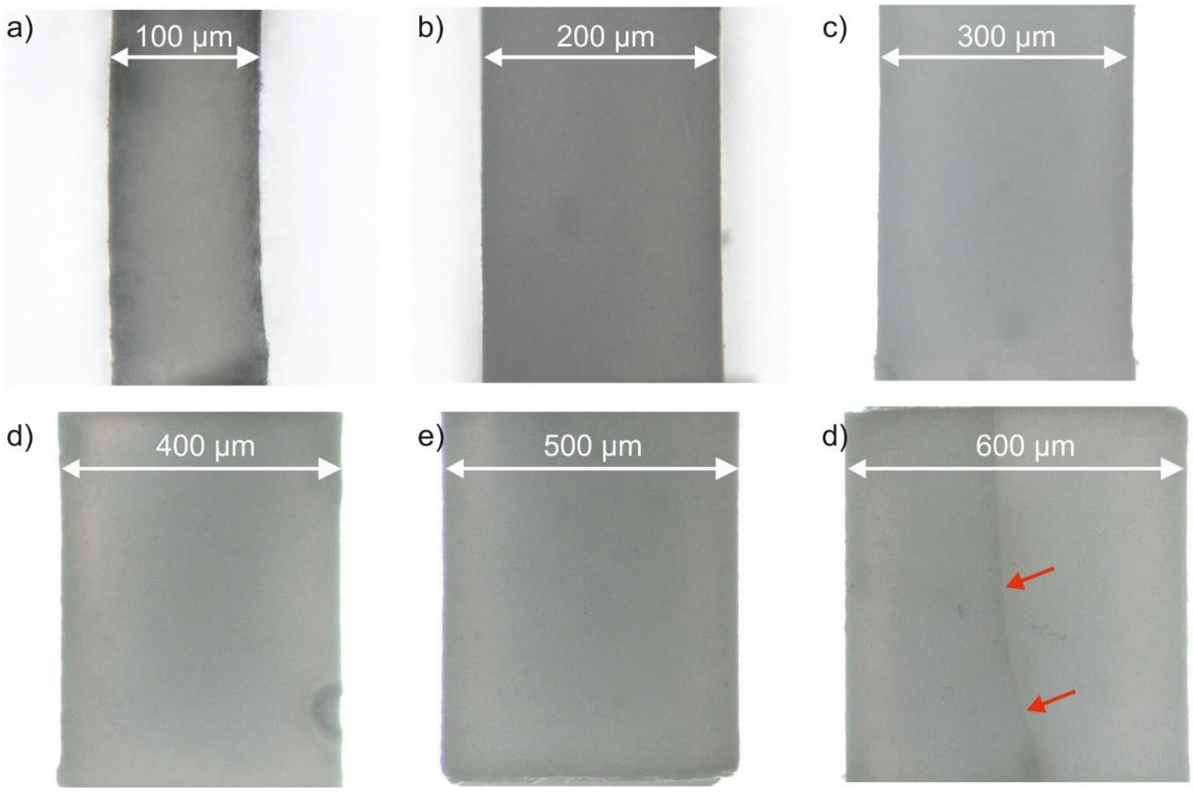

Figure S4. Investigation of the maximum thickness of the printed parts. Printed squares with a thickness of a) 100 µm, b) 200 µm, c) 300 µm, d) 400 µm, e) 500 µm and e) 600 µm are shown after debinding and sintering (1550 °C, 2 h). Samples a-e) could be debinded and sintered without the formation of cracks. Sample d) shows a crack across the square.

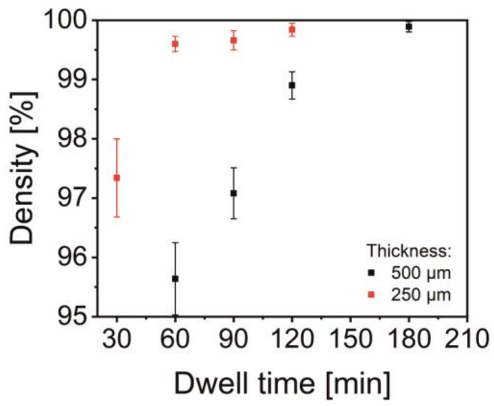

Figure S5. Influence of the sample thickness on pre-densification via sintering at 1550 °C. Samples (3 cm diameter) with a thickness of 250 µm exhibit a density >99 % after a 60 min dwell time. Samples with a thickness of 500 µm require a dwell time higher than 120 min for densification >99 %.

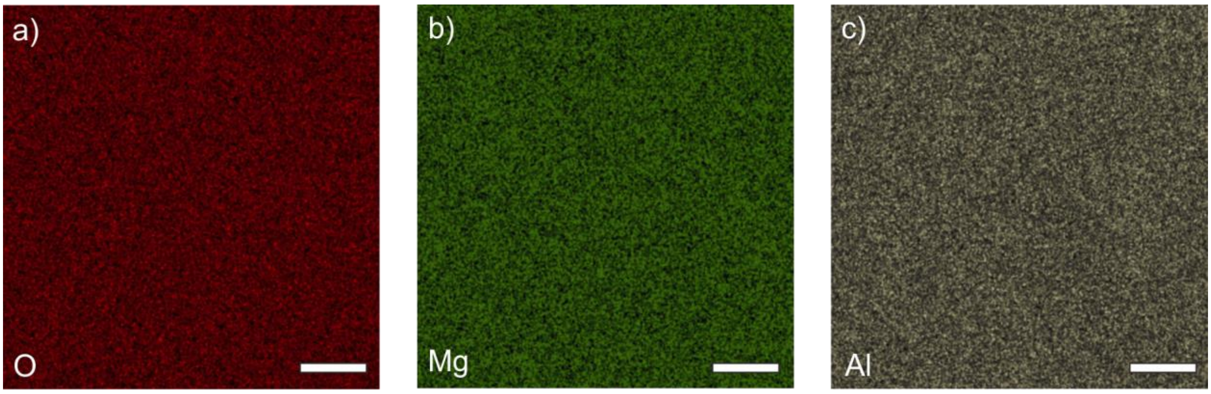

Figure S6. EDX-Mapping of a transparent MAS ceramic. The EDX-measurements reveal a homogenous distribution of a) oxygen, b) magnesium and c) aluminum in the analyzed MAS ceramic after sintering and HIP (scale bars, 5 µm).

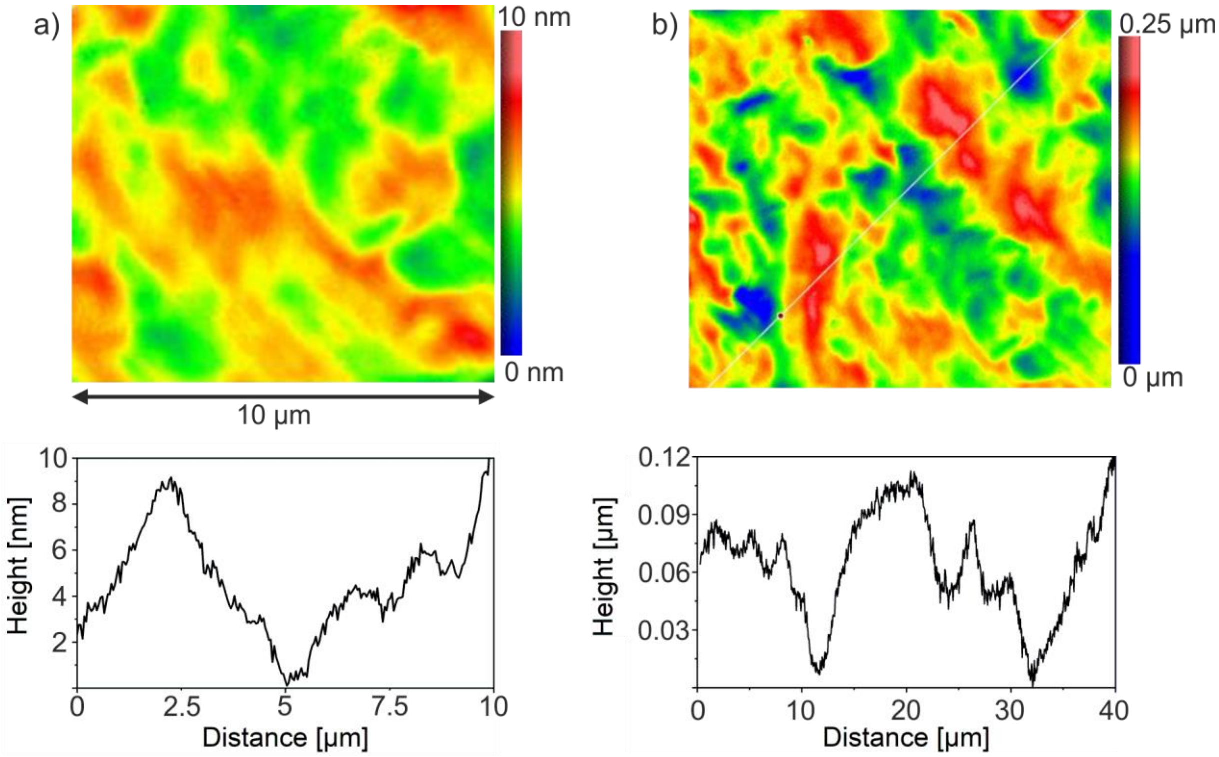

Figure S7. Analysis of the surface roughness of MAS ceramics. a) WLI measurement of a printed sample after HIP (10 × 10 µm). A surface roughness S_q of 10 nm was measured. b) WLI measurement of the same sample on an extended area of 40 × 40 µm. A surface roughness S_q of 26 nm was determined.

Optimization of printing parameters:

In order to find suitable printing parameters, cube-arrays with different parameters were printed and compared with each other. When evaluating the printed cubes, attention was paid to ensure that clear edges were visible and no overpolymerization was observed. Furthermore, it was important to ensure that the printing speed was not too slow (<400 mm s^{-1}) to avoid extending the printing time. A layer thickness of 3 µm was selected, as this is significantly less than the voxel length of around 8 µm (10x objective).

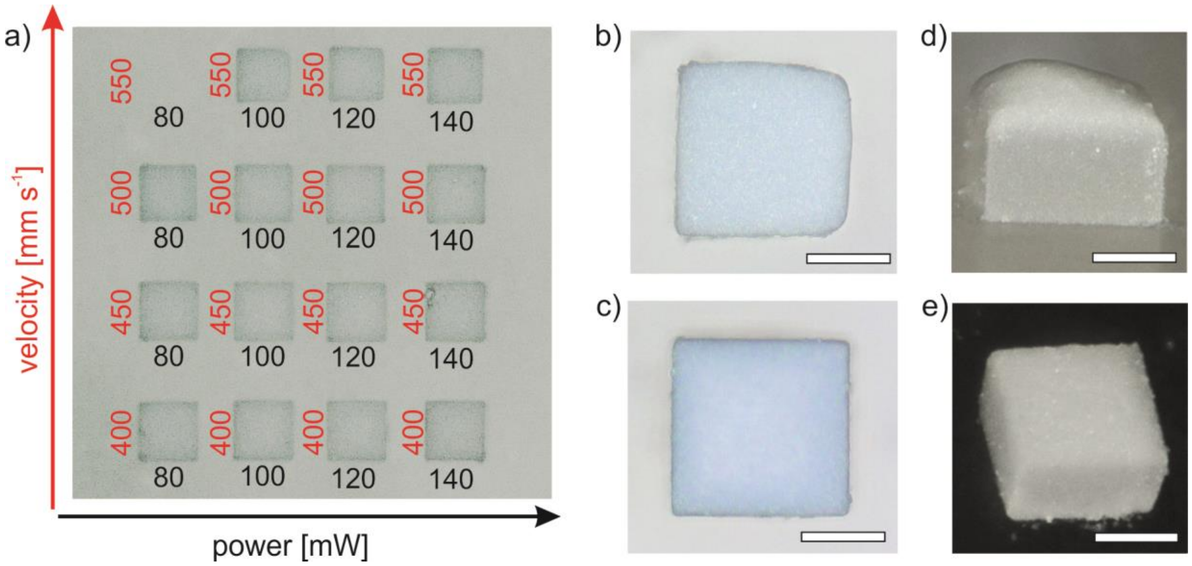

Figure S8. Optimization of printing parameters. a) Printed cube array for the optimization of laser power and scanning speed during TPL. At a power of only 80 mW and a speed of 550 mm s^{-1}, it was no longer possible to print a cube. b) Printed cube (100 mW, 550 mm s^{-1}) which does not have completely defined edges due to insufficient polymerization. c) Printed cube (100 mW, 400 mm s^{-1}) which shows fully formed edges. d) Printed cube (140 mW, 400 mm s^{-1}) which exhibits significant overpolymerization due to excessive exposure. e) Printed cube (100 mW, 400 mm s^{-1}) which shows no overpolymerization (scale bars b-e, 20 µm). The cubes (50 µm diameter) were printed with a layer thickness of 3 µm.

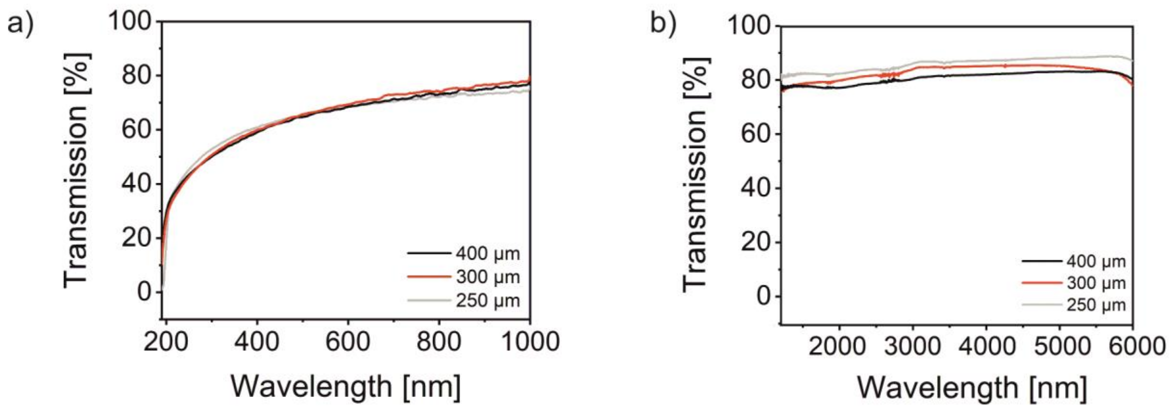

Figure S9. Transmission of MAS ceramics with varying thicknesses. a) UV-Vis measurement of samples with 250 µm, 300 µm and 400 µm thickness. b) FTIR measurement of samples with the same thickness as in a). The transmissions differ only slightly in both the UV-Vis and IR ranges.

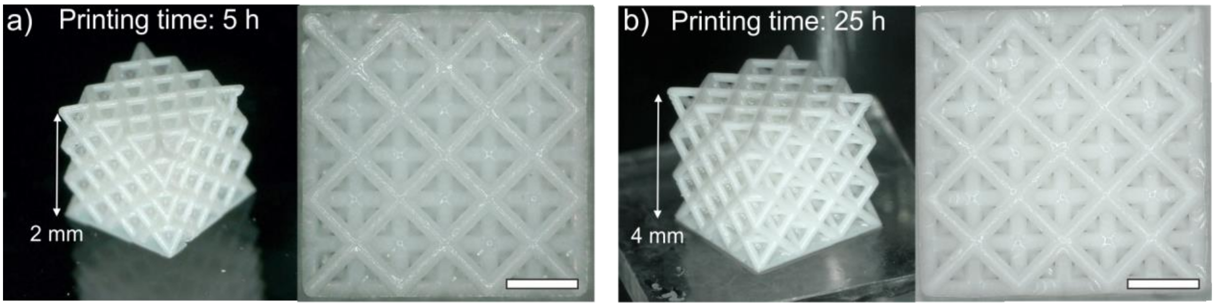

Figure S10. Printing time expenditure related to the printing size. a) Octet lattice cube with a side length of 2 mm (scale bar, 500 µm). The printing time was 5 h. b) Octet lattice cube with a side length of 4 mm (scale bar, 1 mm). The printing time was 5 h. Doubling the print size led to a fivefold increase in printing time.

Table S1. Measurement of the Vickers hardness of transparent MAS ceramics. The measurement was performed with 100 kp and a load duration of 1 s.

No.	HV	GPa
1	1223	11.99
2	1147	11.25
3	1251	12.27
4	1233	12.09
5	1127	11.05
6	1199	11.76
7	1162	11.39
8	1335	13.09
9	1350	13.24
10	1237	12.13
Average	1226	12.03
Standard deviation	70	0.69

Shrinkage during densification:

The isotropic linear shrinkage Y_s can be calculated from the solid loading Φ, the theoretical maximum density ρ_t and the density of the sintered part ρ_s using the following equation:

$$Y_s = 1 - \left(\frac{\Phi}{\rho_s/\rho_t}\right)^{1/3} \quad \text{(S1)}$$
